# Phytochemical Evaluation of Moth Bean (*Vigna aconitifolia* L.) Seeds and Their Divergence

**DOI:** 10.1155/2016/3136043

**Published:** 2016-04-30

**Authors:** Neha Gupta, Nidhi Shrivastava, Pramod Kumar Singh, Sameer S. Bhagyawant

**Affiliations:** ^1^School of Studies in Biotechnology, Jiwaji University, Gwalior 474011, India; ^2^Department of Bioscience & Biotechnology, Banasthali University, Banasthali 304022, India

## Abstract

In the present study, phytochemical contents of 25 moth bean (*Vigna aconitifolia*) seed accessions were evaluated. This includes protease inhibitors, phytic acid, radical scavenging activity, and tannins. The studies revealed significant variation in the contents of theses phytochemicals. Presence of photochemical composition was correlated with seed storage proteins like albumin and globulin. Qualitative identification of total seed storage protein abundance across two related moth bean accessions using two-dimensional gel electrophoresis (2D-GE) was performed. Over 20 individual protein fractions were distributed over the gel as a series of spots in two moth bean accessions. Seed proteome accumulated spots of high intensity over a broad range of pI values of 3–10 in a molecular weight range of 11–170 kDa. In both seed accessions maximum protein spots are seen in the pI range of 6–8.

## 1. Introduction

Plant food extracts and phytochemicals constitute a variety of compounds (most of them are still unexplored) that are part of our diet. In recent years, plant food extracts and phytochemicals have also been highlighted as QSI (quorum sensing inhibitors) [1, 2]. Quorum sensing (QS) is a regulatory mechanism that enables bacteria to make collective decisions with respect to the expression of a specific set of genes which can be related to virulence factors. Pilar et al. [2] summarized the role of plant food extracts and phytochemicals as quorum sensing inhibitors, which have a great potential to inhibit bacterial pathogenesis. In this context, evaluation of plant phytochemicals becomes fundamental.

Phytochemicals are the bioactive compounds that occur naturally in plants. Leguminous seeds are important source of proteins and source of natural antioxidants. Legumes contain a number of phenolic compounds such as flavonoids, phenolic acids, and tannins. There is a considerable interest in finding natural phytochemicals and antioxidants from plants due to their role in the treatment and/or prevention of various diseases. Trypsin inhibitors reduce the incidence of certain cancers and potent anti-inflammatory nature. Several studies have reported on the antioxidant and antiradical activity of tannins. Therefore, characterization of seed proteins vis-à-vis nutritional importance and phytochemical compositions has greatly increased [3].

Moth bean (*Vigna aconitifolia* L.) is a draught resistant legume, belonging to the family* Fabaceae,* commonly grown in arid and semiarid regions of India. It is exceptionally hardy legume and known by various other names including mat bean, matki, Turkish gram, or dew bean. India's driest state, Rajasthan, is the major moth bean growing state contributing almost 86% area of the country [4]. The National Bureau of Plant Genetic resources in New Delhi, India, houses more than 1000 accessions of which cultivars such as RMO-40 and RMO-225 are mostly cultivated in India. Researchers have found that there is a substantial genetic variation between moth bean germplasms.

The literature perusal suggested investigations of pulses as a source of peptides have primarily focused on the major pulses like soybean, chickpea, and mung bean [3]. Characteristics of moth beans with reference to growth and cultivation, total soluble proteins, and nutritional and antioxidant properties of sprout have been investigated [5–7]. Evaluation of phytochemical compositions of the different moth bean accessions remains unexplored and hence scanty. Therefore, present investigation was carried out for phytochemical profiling and qualitative expression of protein abundance across two related moth bean accessions using two-dimensional gel electrophoresis.

## 2. Materials and Methods

### 2.1. Seed Material

A total of 25 moth bean accessions were collected from ICAR-National Bureau of Plant genetic Resources Institute Jodhpur, Rajasthan, India. Seeds were surface-disinfected with 1% (v/v) hypochlorite (0.05% active chloride) for 5 min, rinsed three times in distilled water, dried, and stored in desiccators at room temperature.

### 2.2. Extraction of Seed Storage Proteins

Powdered seed samples were first defeated using chilled acetone and air-dried. For total protein quantification, 100 mg of powdered sample was dissolved in 10 mL of 1 M NaOH. The tubes were incubated overnight followed by centrifugation at 10,000 ×g in cold for 20 min. The supernatant was collected and the total seed protein content was determined by using BSA as a standard [8]. Albumin was isolated following the method of Brown et al. [9]. The pellet, after extraction of water soluble albumin, was further soaked in 1.5 mL chilled 1% (w/v) NaCl solution and kept at 4°C with regular mixing in vortex mixer for 6 hrs. The contents were centrifuged at 12,000 rpm for 10 min to get globulin fraction.

### 2.3. Phytochemical Composition

#### 2.3.1. Determination of Condensed Tannins and Phytic Acid

For tannin isolation, four hundred mg of finely powdered defatted meal was mixed with 40 mL distilled water. The suspension was then boiled for 30 min cooled and subsequently centrifuged at 2000 ×g for 10 min and used as a source for tannin estimation. Tannins were estimated as tannic acid equivalents according to the method of Schandrel [10]. After extraction, 1 mL of the clear supernatant was used as a source of tannins and to this 5 mL of Folin-Denis reagent, 10 mL of sodium carbonate solution was added followed by dilution to 100 mL with water. The tubes were incubated at room temperature for 30 min and the color thus developed was read at 700 nm using Systronics UV-Vis spectrophotometer.

For phytic acid, powdered 50 mg seed samples was extracted overnight in 0.4 mM HCl followed by centrifugation for 20 min at 10,000 ×g at room temperature. Supernatant was collected and used as a source for phytic acid analysis. 10 *μ*L of sample was taken in a microtiter plate, diluted with 90 *μ*L double-distilled water, and followed by addition of 100 *μ*L colorimetric reagent (3 M H_2_SO_4_, 2.5% ammonium molybdate, 10% (w/v) ascorbic acid, and distilled water in 1 : 1 : 1 : 2 ratio). The contents were incubated for 60 min at room temperature and absorbance was taken at 650 nm using Systronics UV-Vis spectrophotometer [11].

#### 2.3.2. Trypsin Inhibitor (TI) Activity

The inhibitor content was measured using* BAPNA* as a substrate [12]. For measuring trypsin inhibitory activity 10 *μ*g of trypsin was mixed with suitable quantity of the sample (to get 50–60% inhibition) and incubated at 25°C before measuring the residual trypsin activity. 10 *μ*L of seed extract was mixed with 80 *μ*L of 50 mM Tris-HCl buffer, pH 8.2, containing 20 mM CaCl_2_, and 10 *μ*L of trypsin and incubated at room temperature at 30 sec interval between two wells on a microtiter plate. The residual activity was measured by adding 125 *μ*L of* BAPNA* (40 mg/mL dimethyl sulfoxide, freshly diluted 1 : 100 in 50 mM Tris-HCl buffer, pH 8.2, and 20 mM CaCl_2_ prewarmed to 37°C) and then incubated at room temperature for 30 min. Reactions were stopped by the addition of 25 *μ*L of 3% (v/v) acetic acid. Liberated* p*-nitroaniline was measured at 410 nm. 100% trypsin activity was measured from the sample minus the inhibitor extract. One unit of trypsin activity was defined as the amount of enzyme which increases the optical density by one unit at 410 nm due to the release of* p*-nitroaniline. One trypsin inhibitor unit was defined as the amount of inhibitor that inhibited 1 unit of trypsin activity [13].

### 2.4. DPPH Radical Scavenging Assay

Scavenging activity on DPPH free radicals by the extracts was assessed according to the method reported by Awah et al. [14] with slight modifications of Gyamfi et al. [15]. Briefly, a 2.0 mL solution of the extract, at different concentrations diluted twofold (2–125 *μ*g/mL) in methanol, was mixed with 1.0 mL of 0.3 mM DPPH in methanol. The mixture was shaken vigorously and allowed to stand at room temperature in the dark for 25 min. Blank solutions were prepared with each test sample solution (2.0 mL) and 1.0 mL of methanol while the negative control was 1.0 mL of 0.3 mM DPPH solution plus 2.0 mL of methanol. L-ascorbic acid was used as the positive control. Thereafter, the absorbance of the assay mixture was measured at 518 nm against each blank with Systronics 2203 UV-Vis spectrophotometer. Lower absorbance of the reaction mixture indicated higher radical scavenging activity.

DPPH radical scavenging activity was calculated using the equation

(1) $$\begin{array}{c} DPPH\%=\left(\;\frac{\left(A_{blank}-A_{sample}\right)}{A_{blank}}\;\right)\times 100. \end{array}$$

### 2.5. 2D-GE Analysis

Proteome analysis of two moth bean accessions revealing the highest concentration of total seed storage proteins was performed by Magni et al. [16]. The defatted moth bean flour was extracted with a solution consisting of 7 M urea, 2 M thiourea, 2% CHAPS, and 65 mM 1,4-dithiothreitol (DTT) in a ratio of 1/30 (w/v) under stirring at room temperature for 2 h. The slurry was centrifuged at 10,000 ×g for 30 min at 4°C and the extracted proteins in the supernatant were analyzed immediately.

The extracted proteins were subjected for 2D-GE analysis as per the standard procedure [17]. The isoelectric focusing (IEF) was performed using 7 cm, pH 3–10 gradient IPG strips (Bio-Rad, USA). The strips were rehydrated overnight in a solution containing of 7 M urea, 2% w/v CHAPS, 15 mM DTT, and 0.5% v/v IPG buffer pH 3–10 (Bio-Rad, USA) containing the protein sample. For the first dimension, 300 *μ*g of protein sample was loaded. These amounts were optimized for the best electrophoretic performance. After 16 h of passive rehydration at 20°C, isoelectric focusing was performed and strips were focused initially at 250 V for 3 h till 8000 volt hours under mineral oil. Strips of IPG were equilibrated for total 25 min prior to SDS-PAGE. After the first dimension, strips were equilibrated for 15 min in the equilibration buffer-I (50 mM Tris-HCl buffer, pH 8.8 containing 6 M urea, 30% w/v glycerol, 2% SDS, and 1% DTT) and then for 10 min in the equilibration buffer-II (equilibration buffer-I containing 4% w/v iodoacetamide instead of DTT). After equilibration, strips were transferred to 12% SDS-PAGE for two-dimensional separation at a constant voltage of 200 V for 3 h. Following electrophoresis, 2D-gels were visualized by staining with colloidal coomassie blue G-250. Protein spots were visualized under white light in a UV transilluminator at 280 nm.

### 2.6. Statistical Analysis

All work was done in triplicate and the data are presented as means ± SD of three independent determinations. Analysis of variance (ANOVA) was carried out using Graph Pad Prism version 5.01. Significance was accepted at *P* ≤ 0.05.

## 3. Results and Discussion

There is a budding interest in characterizing phytochemicals composition of plants like phenolic contents, tannins, trypsin inhibitors, and antioxidants due to their pharmacological significance. This inspiration is because of the structural diversity of natural products that can readily be achieved by chemical synthesis. In the present study, quantitative phytochemical tests demonstrated the presence of trypsin inhibitor; tannins phytic acid; and antioxidant activity and their results were expressed as mean ± standard deviation.

**Figure 1:**
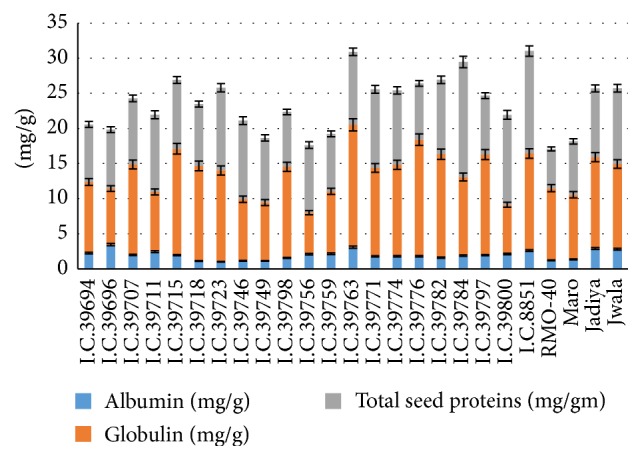
(a) Albumin, (b) globulin, (c) total seed proteins in the seeds of* Vigna aconitifolia*.

The seed storage proteins are nonenzymatic proteins providing nitrogen and sulphur source required during germination and establishment of a new plant [18, 19]. To correlate seed proteins with phytochemicals, albumin and globulin were quantified. Moth bean seeds contain various protein fractions that include albumin and globulin. The highest albumin content of 3.45 mg/g was found in I.C. #39696 and the lowest of 1.01 mg/g in I.C. #39723. The highest globulin content of 17.41 mg/g was observed in I.C. #39763 and the lowest of 5.91 mg/g in I.C. #39756 (Figure 1).

**Figure 2:**
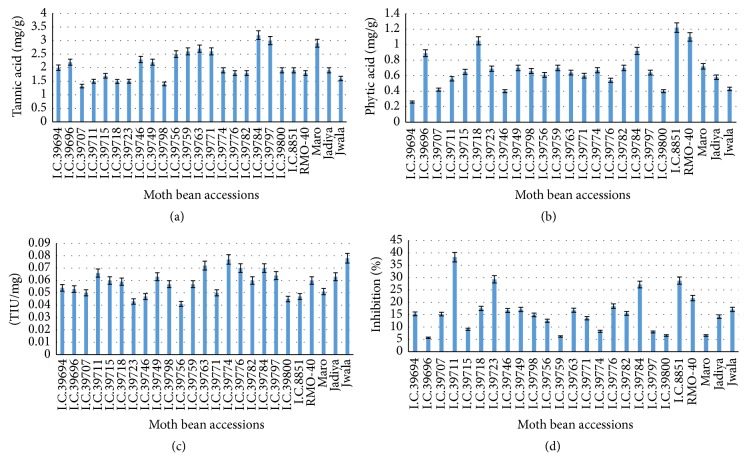
(a) Tannin contents, (b) phytic acid, (c) trypsin inhibitor activity, and (d) free radical scavenging inhibitory activity in the seeds of* Vigna aconitifolia*.

Many tannin-rich seed extracts have been appreciated for their beneficial effects without any obvious toxicity. Plant tannins provide a novel therapeutic option for the treatment of ulcerative colitis. Condensed tannins are located mainly in the testa and play an important role in the defense system of seeds. Their concentration varies depending on several factors such as different genotype, agronomic practices, seasonal variations, and postharvest storage [20]. The total tannin contents in different moth bean accessions were reported as tannic acid equivalents with reference to standard curve. The highest tannin content that was found in cultivar I.C. #39784 is 3.2 mg/g and the lowest concentration that was found in I.C. #39798 is 1.4 mg/g (Figure 2(a)). Such phytochemical investigations of other major legumes have been reported in several studies [21, 22]. Phytic acid is a natural plant oxidant constituting 1–5% of most plant seeds. It usually occurs as phytins having chelating potentials and suppresses iron-catalyzed oxidative reactions, thus enhancing biological activity that can be of therapeutic benefit in treating diseases. The phytic acid concentration varied widely in different moth bean seeds (Figure 2(b)). The highest concentration that was found is of 1.22 mg/g in I.C. #8851 while the lowest of 0.26 mg/g in I.C. #39694.

Trypsin inhibitors were appreciated for reducing the incidence of certain cancers and potent anti-inflammatory properties [3]. Concentration of trypsin inhibitor in the seeds of moth bean is quantified. The highest concentration of trypsin inhibitor that was found in Jwala is 0.078 TIU/mg and the lowest concentration that was found in I.C. #39756 is 0.041 TIU/mg. Differences in the concentration of inhibitor and their activity exist between pulse crop species and may be attributed to varietal differences [23]. Protease inhibitors are suggested as potential drugs for treating various diseases such as human immunodeficiency virus (HIV), hypertension, and neurodegenerative disease, along with various infectious diseases [3]. Most of the research on health benefits of protease inhibitors has been performed with soybean. Soybean extract containing soybean Bowman-Birk inhibitor (BBI) suppressed carcinogenesis in several animal models [24, 25]. However, research in this direction is in progress. Equally to the best of our knowledge there have been no detailed reports confirming the purification and evaluation of trypsin inhibitors from moth bean. A more comprehensive research is needed to screen and analyze the activity of trypsin inhibitors between different accessions of* V. aconitifolia* to understand its therapeutic potential to human health.

Antioxidants are an important part of the defense system of human body and help to cope with oxidative stress caused by reactive oxygen species. There was a significant difference in the antioxidant potentials of different extracts of moth bean cultivars (*P* ≥ 0.05) which represents the variation in percent oxidant scavenging capacity as performed by DPPH free radical scavenging assay. Solvents such as methanol, ethanol, acetone, ethyl acetate, and their combinations have been used by many workers for the extraction of phenolic contents from plant, often with different proportions of water. In the present study, we used methanolic extract, that was found to be more efficient in extraction of lower molecular weight polyphenols [26]. The antioxidant activity exhibited by moth bean seeds varies as shown in Figure 2(d). The highest % inhibition that was observed in I.C. #39711 is 38.26 and the lowest % inhibition that was observed in I.C. #39696 is 5.63 (Figure 2(d)). The present study suggests that free radical scavenging activity of the accessions may be due to the presence of antioxidants, that is, condensed tannins and phytic acids.

Proteins are the actual functional molecules of the cell responsible for almost all the biochemical activities interacting with each other and with a diverse spectrum of other molecules. Many technologies can be used to separate complex proteins; based on charge and mass, one of them includes PAGE. One-dimensional technique like PAGE does not provide enough resolving power. Now protein separation technology pushed to its limits [27]. The ultimate goal is to resolve all the individual proteins in the cell. Such dominating proteomic technique of fractionation includes two-dimensional gel electrophoresis, which is also known as ISO-DALT (isoelectric focusing and Dalton, the unit of protein mass).

In the present analysis, 2D-GE was employed to achieve separation on the basis of charge alone and on the basis on mass alone. Isoelectric focusing is used in the first dimension followed by SDS-PAGE in the second dimension. Seed storage proteins were separated in the orthogonal dimension. After nonselective staining, the two-dimensional protein profiles of moth bean accessions were generated in the form of spots. Over 20 individual protein fractions were distributed over the gel in two different moth bean accessions as a series of spots, which represents approximately 20% of moth bean proteome. Such studies are also performed in other legumes like pea [28].

**Figure 3:**
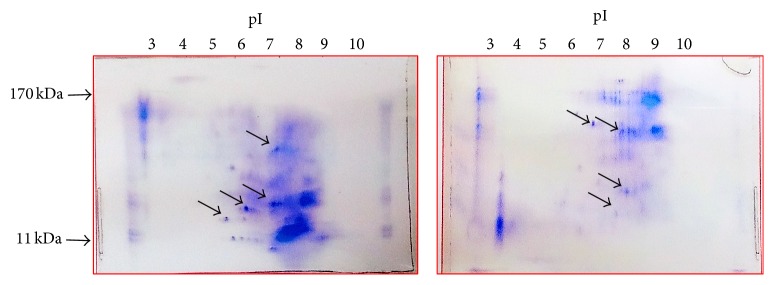
2D-GE pattern of I.C. #39784 and I.C. #8851 moth bean accessions.

Total seed storage protein extract from mature dry* V. aconitifolia* seeds was showing specific two-dimensional gel patterns with significant variation in a more molecular weight with range of 11 kDa–170 kDa. In both seed accessions maximum protein spots are seen in the pI range of 6–8 (Figure 3). The map denotes the intrinsically complex pattern of moth bean total seed storage proteins with several spots of the same molecular weight and different pIs, suggesting charge heterogeneity. Such diversity may be generated by different posttranslational modifications. The data produced in the present 2D-GE experiment are visual in nature. Some of the polymorphic spots are shown by arrow in Figure 3. Further investigations include downstream analysis vis-à-vis capturing the images of protein spots from the stained 2D-gels, isolating spots and submitting them for mass spectrometry. Such analysis will provide the technology platform for high-throughput protein identification in moth bean. Screening and characterization of the purified phytochemicals by preparatory separation methods like LC-MS are essential to separate individual phytochemicals from less studied moth bean seeds. Further research is needed to improve our knowledge regarding bioactive capacities and cytotoxicity effects of these phytochemicals to evaluate therapeutic effects of moth bean.
